# The use of triple therapy, ICS/LABA/LAMA, in the treatment of asthma: A real-life Latin-American specialized centers experience

**DOI:** 10.1016/j.jacig.2025.100606

**Published:** 2025-11-14

**Authors:** Ricardo Martinez-Tenopala, Victor Gonzalez-Uribe, Carlos Andres Gomez-Nuñez, Jimena Prieto-Gomez, Maria Julia Rendon-Salazar, Tamara Hernandez-Hernandez, Cesar F. Pozo Beltran, Christian R. Alcocer-Arreguin, Paola de Baro Alvarez, Zaira S. Mojica-Gonzalez

**Affiliations:** a AlergiaMx, Benito Juarez, Mexico City, Mexico; b Facultad Mexicana de Medicina, Universidad La Salle Mexico, Tlalpan, Mexico City, Mexico; c Subdireccion de Enseñanza Y Calidad de La Secretaria de Salud de Baja California Sur, La Paz, Mexico; d Sin Alergia GDL, Guadalajara, Jalisco, Mexico; e Pathology & Immunohistochemistry Department, Hospital General de Mexico Dr. Eduardo Liceaga, Cuauhtemoc, Mexico City, Mexico

**Keywords:** Asthma, triple therapy, LAMA, asthma management

## Abstract

**Background:**

The use of triple therapy with inhaled corticosteroids (ICSs), long-acting beta-agonists (LABAs), and long-acting muscarinic antagonists (LAMAs) is increasingly common in asthma management, particularly in patients with uncontrolled disease. However, real-world data from Latin-American settings are limited.

**Objective:**

We sought to compare asthma control and exacerbation frequency among patients receiving ICS/LABA/LAMA versus ICS/LABA alone at steps 4 and 5 of the Global Initiative for Asthma guidelines in specialized centers in Mexico.

**Methods:**

A cross-sectional observational study was conducted in 3 allergy and pulmonary clinics. Adult patients with asthma receiving maintenance therapy at Global Initiative for Asthma step 4 or 5 were recruited. Demographic, clinical, and spirometric data were collected. Asthma control was assessed using the asthma control test. Comparisons were made between patients receiving ICS/LABA/LAMA and those receiving ICS/LABA only. Ordinal logistic regression was used to identify predictors of asthma control.

**Results:**

Among 279 patients (71% female; mean age, 51.4 ± 12.3 years), 79 received triple therapy. At step 4, ICS/LABA/LAMA users had better asthma control than those on ICS/LABA alone (*P* = .03). At step 5, ICS/LABA/LAMA users showed lower asthma control test scores and more frequent exacerbations (*P* < .05), likely reflecting greater baseline severity. At step 4, ICS/LABA/LAMA use was associated with better asthma control in the unadjusted analysis; however, this association was no longer statistically significant after adjustment in the ordinal logistic regression model.

**Conclusions:**

ICS/LABA/LAMA may improve asthma control at step 4 but not at step 5, possibly due to more severe disease and previous use of biologics in the latter group. These findings highlight the need for tailored therapy and better phenotyping in severe asthma.

Asthma is one of the most common chronic respiratory diseases, affecting all age groups, and is the most common in children.^1^ The goal in the treatment of asthma is to achieve long-term symptom control and minimize the risk of exacerbations, improve lung function, and not require oral corticosteroids (OCSs) in maintenance; however, patients with asthma have inadequate control in approximately 54% to 57% of the cases and high exacerbation rates in patients with moderate to severe disease (Global Initiative for Asthma [GINA] steps 4 and 5).^2^^,^^3^

Long-acting muscarinic antagonists (LAMAs) have a different mechanism of bronchodilation than long-acting beta-agonists (LABAs), which is why these are used as add-on therapy in asthma that persists uncontrolled.^1^ LAMAs antagonize the action of acetylcholine by inhibiting muscarinic M1 and M3 receptors in the airways, resulting in smooth muscle relaxation, reduced airway inflammation, decreased mucus secretion, and prevention of asthma-related airway remodeling.^4^^,^^5^ The inhaled corticosteroid (ICS) plus LABA plus LAMA (ICS/LABA/LAMA) combination has been shown to be effective in counteracting the inflammatory response by inhibiting IL-4, IL-5, IL-6, IL-9, IL-13, TNF-α, and thymic stromal lymphopoietin.^2^ In this context, it is allowed to reduce the concentration of a single agent to obtain the same relaxing effect.

Current GINA guidelines recommend consideration of LAMA in patients with asthma that remain uncontrolled despite medium- or high-dose ICS/LABAs in patients with moderate to severe asthma.^1^^,^^2^^,^^6^^,^^7^ In step 4, LAMAs are indicated as an alternative triple controller therapy, whereas in step 5 these are indicated as the preferred controller; however, the efficacy and adverse effects of adding LAMAs are controversial concerning disease control, improving quality of life, and lung function.^6^^,^^8^, ^9^, ^10^, ^11^ In particular, LAMA as an add-on therapy has been recommended regardless of the baseline asthma phenotype, without any specific guidance on which patient groups should receive LAMA.^6^ However, limited data exist on the frequency and characteristics of patients with asthma using LAMA.

In this context, the objectives of this study were to determine the frequency of triple therapy (ICS/LABA/LAMA) use and to characterize the sociodemographic and clinical characteristics of patients receiving triple therapy at 3 asthma monitoring centers, which specialize in the management of severe asthma and serve a large and diverse patient population. In addition, we aimed to identify any distinguishing features between patients using LAMA and those treated with step 4 and step 5 therapies but without LAMA. This study seeks to provide additional information on the indications of ICS/LABA/LAMA in the treatment of patients with severe asthma.

## Methods

### Study design and study population

This was an observational, descriptive, cross-sectional study. Data were collected from medical records of all patients with asthma who had been followed up in 3 asthma follow-up clinics. The centers are leading referral institutions in the country, with extensive experience in asthma diagnosis and management. Patients are examined every weekday, with a total of 700 registered patients and approximately 100 new cases annually. A physician is assigned to the asthma outpatient clinic in which 3 professors provide supervision and consultation. Patients with asthma receiving GINA step 4 or step 5 treatment and with at least 1-year follow-up in our center were included after obtaining written consent. It was also noted whether the patients were using ICS/LABA/LAMA or not. To consider patients as “using LAMA,” patients should have been on ICS/LABA/LAMA for at least 6 months. Most patients were insured under Mexico’s universal public health care system (Mexican Institute of Social Security or Institute of Health for Wellbeing) or private health insurance. Medication adherence was not systematically recorded in the medical records and is acknowledged as a limitation.

The only LAMA used in the study was glycopirronium, because it was the only ICS/LABA/LAMA aerosol spray device approved and available in our country before 2023. Although newer fixed-dose LAMA-containing dry powder inhaler combinations have since become available, there were not included in this analysis to maintain consistency in treatment comparisions.

### Clinical data collection

The file review phase of the study was conducted from June 2023 to June 2024. During this period, the records of patients who had been followed up at the referral centers for at least 1 year were reviewed. The data collected included the sociodemographic features of the patients, phenotypic features and clinical presentations of asthma, presence of allergic and systemic comorbidities, and asthma treatment steps. Clinical follow-up parameters including asthma control test (ACT) scores, number of asthma exacerbations and hospitalizations in the previous year, and pulmonary function test results were also included. In addition, we evaluated the use of ICS/LABA/LAMA (budesonide/glycopirronium/formoterol), the treatment step at which LAMA was initiated, and the current treatment to which LAMA was added. Following data evaluation, we documented the characteristics of the patients using ICS/LABA/LAMA, as well as their disease features. The outcomes of the patients who did and did not receive LAMA at step 4 and step 5 were compared within each step. For each patient, the most recent ACT score and spirometry values (minimum and maximum FEV_1_) over the 1-year observation period were used. Hospitalizations were documented from clinical records rather than patient self-report.

### Definitions

#### Phenotypic evaluation

*Allergic.* Patients sensitized to at least 1 inhalant allergen consistent with their history and clinical features in a skin prick test and/or specific IgE measurement.
*Eosinophilic:* Patients with a blood eosinophil count of 300/μL or higher during an OCS-free period or 150/μL or higher under OCS at least twice during the follow-up period.
*Noneosinophilic:* Patients whose blood eosinophil count did not reach 300/μL in the OCS-free period or 150/μL under OCS in at least 3 separate measurements.

Phenotypic groups were classified as follows: allergic eosinophilic, nonallergic eosinophilic, allergic no-eosinophilic, and nonallergic no-eosinophilic.

#### Based on age at asthma onset

*Early-onset asthma:* Asthma diagnosed before the age of 18 years.
*Adult-onset asthma:* Asthma diagnosed between the age of 18 and 40 years.
*Late-onset asthma:* Asthma diagnosed after the age of 40 years.

#### Asthma control status at the last visit

Patients were classified on the basis of following criteria: (1) having an ACT score of 20 or higher at the last visit, (2) FEV_1_ variability of less than 12% between visits in the past year, and (3) having no history of asthma exacerbation in the previous year.

Well-controlled patients had to meet all 3 criteria, partial-controlled patients did not meet 1 or 2 criteria, and patients with uncontrolled asthma did not meet any of the criteria.

#### Asthma exacerbation

Asthma exacerbation was defined as a worsening of asthma symptoms requiring systemic steroid use for at least 3 days.

#### Pulmonary function test

*Minimum FEV*_*1*_: Lowest FEV_1_ value during follow-up.
*Maximum FEV*_*1*_: Highest FEV_1_ value during follow-up.

### Measurements

#### Skin prick test

Skin prick tests were performed using common aeroallergen extracts (*Dermatophagoides pteronyssinus*; *Dermatophagoides farinae*; grass pollens, weed pollens, tree pollens, cereal pollens, molds, and cat and dog epithelia) (ALK-DIEMSA, Mexico City, Mexico).

Prick test results were considered positive if edema at least 3 mm or more accompanied by erythema occurred at the 15-minute reading, validated by a positive control (histamine 10 mg/mL) and a negative control (saline).

#### Specific IgE

The ImmunoCAP fluoroenzyme immunoassay system (Phadia, Uppsala, Sweden) was used for specific IgE testing. Values equal to or higher than 0.35 kU/L were defined as positive.

#### Pulmonary function test

FEV_1_, forced vital capacity, peak expiratory flow, and maximum midexpiratory flow were measured using a spirometry device (ZAN 100; nSpire Health GmbH, Oberthulba, Germany) and evaluated according to the American Thoracic Society/European Respiratory Society guidelines.

### Statistical analyses

**Fig 1:**
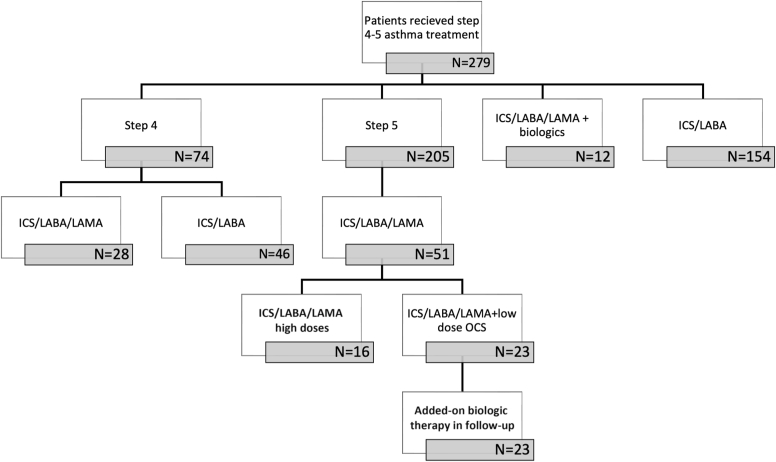
Flowchart of the study.

Statistical analyses were performed using SPSS version 23 software (SPSS, Chicago, Ill). An ordinal logistic regression model was used to assess the association between clinical variables and asthma control status, categorized as well controlled, partially controlled, and uncontrolled. Independent variables included LAMA use, GINA treatment level, FEV_1_ (% predicted), body mass index (BMI), eosinophilic phenotype, age, and sex. For step 5 patients, biologic use (dupilumab, mepolizumab, tezepelumab) was recorded and considered as a separate subgroup in descriptive analyses. Biologic therapy was therefore reported descriptively (Fig 1 and Table IV) but was not included in the regression model, because comparative analysis of biologics was beyond the objective of this study.

The normality of distribution was assessed using the Kolmogorov-Smirnov test. Frequency of distribution was analyzed, and descriptive statistics were calculated using mean and SD for normally distributed variables, and median and interquartile range for non–normally distributed variables. Categorical variables were compared using Fisher exact test or Pearson chi-square test, as appropriate. Comparisons between groups were performed using Student *t* test/Mann-Whitney *U* test and 1-way ANOVA. *P* values less than .05 were considered statistically significant.

### Institutional review board statement

This study was approved by the Institutional Review Board of Universidad La Salle Mexico, and the protocols used in the study were approved by the Committee of Human Subjects Protection & BioEthics of Universidad La Salle Mexico, Mexico City and Instituto Jaliscience de Investigación Clínica A.C. with registrations and approvals in December 2022 and January 2023.

A total of 279 patients were included in this observational study conducted from June 2023 to June 2024, after approval from the institutional ethics committee.

## Results

In total, 279 patients with asthma received step 4 (26.5%, n = 74) and step 5 (73.5%, n = 205) treatment, with at least 1 year of follow-up in the 3 clinics. The mean age was 50.84 ± 12.42 years, and 77.1% (n = 215) were females. A total of 79 (28.3%) patients (female/male: 60/19), with a mean age of 52.45 ± 11.61 years, used LAMA in a budesonide/glycopirronium/formoterol combination therapy (Fig 1). All patients had regular follow-up, with a median of 5 visits per year (range, 1-15). The median follow-up period in our centers was 3 years (minimum-maximum, 1-9). Most patients were overweight (39.2%, n = 31) or obese (43.0%, n = 34) and had adult-onset (53.2%, n = 42) or late-onset (38.0%, n = 30) asthma. More than half were nonallergic (64.6%, n = 51) and eosinophilic (70.9%, n = 56). The mean ACT score at the last visit was 20.88 ± 4.70, and more than half of the patients were either well controlled (34.2%, n = 27) or partially controlled (32.9%, n = 26) in the previous year (Table I). The most common allergic comorbidities were allergic rhinitis (30.7%, n = 24), nonsteroidal anti-inflammatory drug–exacerbated respiratory disease (16.5%, n = 13), food allergy (5.1%, n = 4), and urticaria (5.1%, n = 4). The most common systemic comorbidities were chronic rhinosinusitis (34.2%, n = 27), nasal polyposis (25.3%, n = 20), gastroesophageal reflux (19.0%, n = 15), hypertension (13.9%, n = 11), and diabetes mellitus (8.9%, n = 7).

**Table I:** Demographic features of the patients who used LAMAs (n = 79)

Characteristic	Value	% (n)
Sex, % (n)	Female	75.9 (60)
	Male	24.1 (19)
Age (y), mean ± SD	52.45 ± 11.61	
Allergy, % (n)	Allergic	35.4 (28)
	Nonallergic	64.6 (51)
Blood eosinophilia, % (n)	Eosinophilic (≥300 cell/μL)	70.9 (56)
	Noneosinophilic (<300 cells/μL)	29.1 (23)
Phenotype, % (n)	AE	25.3 (20)
	ANE	8.9 (7)
	NAE	44.3 (35)
	NANE	21.5 (17)
Obesity, % (n)	Obese (BMI ≥ 30)	43.0 (34)
	Overweight (BMI = 25-29.9)	39.2 (31)
	Normal (BMI < 25)	17.7 (14)
BMI, mean ± SD	29.74 ± 5.74	
Age of asthma onset (y), mean ± SD	35.16 ± 12.04	
Asthma onset, % (n)	Early onset	8.9 (7)
	Adult onset	53.2 (42)
	Late onset	38.0 (30)
Disease duration (y), median (min-max)	17 (1-34)	
Follow-up duration (y), median (min-max)	3 (1-9)	
History of smoking, % (n)	Nonsmoker	68.4 (55)
	Smoker	7.6 (6)
	Ex-smoker	24.1 (18)
Asthma control, % (n)	Well	34.2 (27)
	Partial	36.7 (29)
	Uncontrolled	29.1 (23)
ACT score, mean ± SD	20.88 ± 4.70	

*AE*, Allergic eosinophilic; *ANE*, allergic no-eosinophilic; *min*, minimum; *max*, maximum; *NAE*, nonallergic eosinophilic; *NANE*, nonallergic no-eosinophilic.

### LAMA in step 4 patients

LAMA was initiated more frequently in patients receiving step 4 treatment than in those on step 5 treatment (n = 28 [37.8%] vs n = 51 [24.8%]) (*P* = .034). LAMA was used in a triple-therapy combination (budesonide/glycopirronium/formoterol) (ICS/LAMA/LAMA) at step 4 in 28 patients, constituting 37.8% of step 4 patients and 35.4% of all patients using ICS/LABA/LAMA (Fig 1). The mean age was 54.05 ± 10.45 years, and 89.3% (n = 25) were female. More than half of the patients were eosinophilic (57.1%, n = 16), and most were nonallergic (71.4%, n = 20). The nonallergic eosinophilic group was the most common (39.3%, n = 11), followed by the nonallergic noneosinophilic group (35.7%, n = 10).

When comparing patients with LAMA (n = 28) and without LAMA (n = 46) in step 4, there was no significant difference in terms of age, sex, being eosinophilic or allergic, BMI, asthma onset, history of smoking, and number of asthma exacerbations in the previous year (Table II). Although the nonallergic eosinophilic and nonallergic no-eosinophilic groups were more prevalent among LAMA users, the distribution of phenotypes was more homogeneous in non-LAMA users. However, there was no statistical difference between the 2 groups regarding phenotypic distribution (*P* = .082). Asthma control scores at the last visits were similar in both groups (*P* = .088), but when we looked at the control status in the previous year, patients who received ICS/LABA/LAMA were more likely to be well controlled than those who did not (*P* = .001). Patients who received ICS/LABA/LAMA had lower minimum FEV_1_ values during the follow-up (*P* = .030).

**Table II:** Comparison of the features of patients with and without LAMA in step 4

Features		With ICS/LABA/LAMA (n = 28)	Without add-on LAMA (n = 46)	*P* value
Age (y), mean ± SD		54.05 ± 10.45	51.09 ± 12.35	.290
Sex: female, % (n)		89.3 (25)	91.3 (42)	.999
Allergy, % (n)	Allergic	28.6 (8)	45.7 (21)	.225
	Nonallergic	71.4 (20)	54.3 (25)	
Blood eosinophilia, % (n)	Eosinophilic (≥300 cell/μL)	57.1 (16)	39.1 (18)	.205
	Noneosinophilic (<300 cells/μL)	42.9 (12)	60.9 (28)	
Phenotype, % (n)	AE	17.9 (5)	15.2 (7)	.082
	ANE	7.1 (2)	30.4 (14)	
	NAE	39.3 (11)	23.9 (11)	
	NANE	35.7 (10)	30.4 (14)	
Obesity, % (n)	Obese (BMI ≥ 30)	46.4 (13)	58.7 (27)	.451
	Overweight (BMI = 25-29.9)	32.1 (9)	19.6 (9)	
	Normal (BMI < 25)	21.4 (6)	21.7 (10)	
BMI		29.60 ± 5.65	30.96 ± 6.14	.344
The onset age of asthma (y), mean ± SD		37.17 ± 11.90	34.54 ± 10.58	.325
Asthma onset, % (n)	Early onset	7.1 (2)	6.5 (3)	.779
	Adult onset	57.1 (16)	65.2 (30)	
	Late onset	35.7 (10)	28.3 (13)	
History of smoking, % (n)	Nonsmoker	71.4 (20)	69.6 (32)	.154
	Smoker	17.9 (5)	6.5 (3)	
	Ex-smoker	10.7 (3)	23.9 (11)	
Asthma control	Well	42.9 (12)	54.3 (25)	.001
	Partial	32.1 (9)	2.2 (1)	
	Uncontrolled	25.0 (7)	43.5 (20)	
ACT score, mean ± SD		20.50 ± 4.54	22.11 ± 3.42	.088
No. of visits per year, median (min-max)		4 (1-13)	3 (1-5)	<.001
No. of asthma exacerbations, median (min-max)		0 (0-3)	0 (0-2)	.164
Pulmonary function tests, mean ± SD	FEV_1_min %	70.15 ± 19.18	78.26 ± 20.30	.101
	FEV_1_min mL	1.57 ± 0.59	1.91 ± 0.65	.030
	FEV_1_/FVC min	69.00 ± 10.58	72.43 ± 11.29	.209
	FEV_1_max %	84.44 ± 17.43	92.04 ± 19.43	.099
	FEV_1_max mL	1.91 ± 0.74	2.22 ± 0.64	.065
	FEV_1_/FVC max	74.07 ± 8.33	76.50 ± 8.79	.250

*AE,* Allergic eosinophilic; *ANE*, allergic no-eosinophilic; *FEV*_*1*_*min*, minimum FEV_1_; *FEV*_*1*_*max*, maximum FEV_1_; *FVC*, forced vital capacity; *min*, minimum; *max*, maximum; *NAE*, nonallergic eosinophilic; *NANE*, nonallergic no-eosinophilic.

### LAMA in step 5 patients

A total of 51 patients received ICS/LABA/LAMA at step 5, representing 24.8% of the patients who received step 5 treatment and 64.6% of all patients using LAMA. In 16 patients, ICS/LABA/LAMA was prescribed in high dose of ICS, and in 23 patients was added to high-dose ICS ± OCS, but these patients eventually switched to biologic therapy. The remaining 12 patients were on biologic therapy in addition to high-dose ICS/LABA/LAMA ± OCS (Fig 1). Among step 5 patients, when comparing those with and without LAMA (n = 51 and n = 154, respectively), there were no significant differences in terms of age, sex, blood eosinophil count, atopy, asthma phenotypes, BMI, age of asthma onset, and smoking history (Table III). ACT scores were similar between both groups (*P* = .362). Patients with ICS/LABA/LAMA at step 5 experienced more frequent asthma exacerbations than those without LAMA (*P* < .001) and therefore being well controlled was more common in patients without add-on LAMA, whereas the partially controlled rate was higher among ICS/LABA/LAMA users (*P* < .001). The patients with ICS/LABA/LAMA had significantly lower pulmonary function test values than those without add-on LAMA (*P* < .001). The mean minimum FEV_1_ of patients with ICS/LABA/LAMA was 1.38 ± 0.55 L (56.49% ± 20.52%), whereas it was 1.99 ± 0.75 L (75.69% ± 18.31%) for patients without LAMA. The mean maximum FEV_1_ value of patients with add-on LAMA was 1.85 ± 0.67 L (72.55% ± 25.52%) and was 2.48 ± 0.86 L (93.81% ± 18.40%) in the patients without add-on LAMA (Table III).

**Table III:** Comparison of the features of patients with and without add-on LAMA in step 5 (n = 205)

Features		With ICS/LABA/LAMA (n = 51)	Without add-on LAMA (n = 154)	*P* value
Age (y), mean ± SD		52.16 ± 12.18	49.74 ± 12.80	.239
Sex: female, % (n)		68.6 (35)	73.4 (113)	.634
Allergy, % (n)	Allergic	39.2 (20)	52.6 (81)	.135
	Nonallergic	60.8 (31)	47.4 (73)	
Blood eosinophilia, % (n)	Eosinophilic (≥300 cell/μL)	78.4 (40)	75.3 (116)	.794
	Noneosinophilic (<300 cells /μL)	21.6 (11)	24.7 (38)	
Phenotype, % (n)	AE	29.4 (15)	37.0 (57)	.374
	ANE	9.8 (5)	15.6 (24)	
	NAE	47.1 (24)	38.3 (59)	
	NANE	13.7 (7)	9.1 (14)	
Obesity, % (n)	Obese (BMI ≥ 30)	43.1 (22)	42.9 (66)	.126
	Overweight (BMI = 25-29.9)	41.2 (21)	27.9 (43)	
	Normal (BMI < 25)	15.7 (8)	29.2 (45)	
BMI, mean ± SD		29.59 ± 5.76	29.07 ± 6.24	.605
The onset age of asthma (y), mean ± SD		35.27 ± 12.68	33.94 ± 12.21	.504
Asthma onset, % (n)	Early onset	9.8 (5)	10.4 (16)	.826
	Adult onset	51.0 (26)	55.2 (85)	
	Late onset	39.2 (20)	34.4 (53)	
History of smoking, % (n)	Nonsmoker	68.6 (35)	81.2 (125)	.169
	Smoker	2.0 (1)	1.9 (3)	
	Ex-smoker	29.4 (15)	16.9 (26)	
Asthma control	Well	34.0 (17)	55.2 (85)	<.001
	Partial	34.0 (17)	7.1 (11)	
	Uncontrolled	32.0 (16)	37.7 (58)	
ACT score, mean ± SD		21.33 ± 4.72	21.95 ± 3.96	.362
No. of visits per year, median (min-max)		6 (2-15)	8.5 (0-24)	.111
No. of asthma exacerbations, median (min-max)		1 (0-6)	0 (0-5)	<.001
Pulmonary function tests, mean ± SD	FEV_1_min %	56.49 ± 20.52	75.69 ± 18.31	<.001
	FEV_1_min mL	1.38 ± 0.55	1.99 ± 0.75	<.001
	FEV_1_/FVC min	64.17 ± 12.23	72.45 ± 9.02	<.001
	FEV_1_max %	72.55 ± 25.52	93.81 ± 18.40	<.001
	FEV_1_max mL	1.85 ± 0.67	2.48 ± 0.86	<.001
	FEV_1_/FVC max	68.56 ± 12.19	76.69 ± 7.60	<.001

*AE,* Allergic eosinophilic; *ANE*, allergic no-eosinophilic; *FEV*_*1*_*min*, minimum FEV_1_; *FEV*_*1*_*max*, maximum FEV_1_; *FVC*, forced vital capacity; *min*, minimum; *max*, maximum; *NAE*, nonallergic eosinophilic; *NANE*, nonallergic no-eosinophilic.

The patients were divided into 3 groups (group 1, group 2, and group 3) according to the treatment stage at which LAMA was added in step 5. In group 1 (n = 16), ICS/LABA/LAMA was prescribed at a high dose with reference to ICS. In group 2 (n = 23), ICS/LABA/LAMA was prescribed at a high dose with reference to ICS and/or low-dose OCS, and biologic treatment was added during follow-up. In group 3 (n = 12), ICS/LABA/LAMA was prescribed at a high dose with reference to ICS while patients were already receiving biologic treatment and/or low-dose OCS.

The proportion of eosinophilia was higher in groups 2 and 3 than in group 1 (*P* = .003). Although nonallergic eosinophilic was more frequent in groups 2 and 3 (n = 14, 60.9% and n = 7, 58.3%, respectively), almost half of group 1 was nonallergic no-eosinophilic (n = 7, 43.8%) (*P* = .002). Group 2 had the highest number of asthma exacerbations (*P* = .041). Minimum FEV_1_ was lower in group 3 than in both group 1 and group 2 (1.04 ± 0.34 L vs 1.32 ± 0.50 L and 1.58 ± 0.59 L, respectively) (*P* = .018). Similarly, the maximum FEV_1_ was lowest in group 3 compared with group 1 and group 2 (1.56 ± 0.59 L vs 1.64 ± 0.53 L and 2.14 ± 0.69 L, respectively) (*P* = .014) (Table IV).

**Table IV:** Comparison of the features of the patients using ICS/LABA/LAMA in step 5 (n = 51)

Features		Group 1 (n = 16)	Group 2 (n = 23)	Group 3 (n = 12)	*P* value
Age (y), mean ± SD		54.31 ± 10.73	49.65 ± 13.33	54.92 ± 11.79	.369
Sex: female, % (n)		75.0 (12)	65.2 (15)	66.7 (8)	.795
Allergy, % (n)	Allergic	37.5 (6)	39.1 (9)	41.7 (5)	.975
	Nonallergic	62.5 (10)	60.9 (14)	58.3 (7)	
Blood eosinophilia, % (n)	Eosinophilic (≥300 cell/μL)	50.0 (8)	95.7 (22)	83.3 (10)	.003
	Noneosinophilic (<300 cells /μL)	50.0 (8)	4.3 (1)	16.7 (2)	
Phenotype, % (n)	AE	15.4 (4)	35.7 (8)	25.0 (3)	.001
	ANE	12.5 (2)	4.3 (1)	16.7 (2)	
	NAE	18.8 (3)	60.9 (14)	58.3 (7)	
	NANE	43.8 (7)	00.0 (0)	00.0 (0)	
Obesity, % (n)	Obese (BMI ≥ 30)	56.3 (9)	30.4 (7)	41.7 (5)	.492
	Overweight (BMI = 25-29.9)	37.5 (6)	47.8 (11)	41.7 (5)	
	Normal (BMI < 25)	6.3 (1)	21.7 (5)	16.7 (2)	
BMI, mean ± SD		31.85 ± 7.02	28.34 ± 4.57	29.13 ± 5.97	.174
The onset age of asthma (y), mean ± SD		35.31 ± 11.25	32.21 ± 11.96	34.41 ± 11.28	.761
Asthma onset, % (n)	Early onset	0.0 (0)	17.4 (4)	8.3 (1)	.243
	Adult onset	56.3 (9)	52.2 (12)	41.7 (5)	
	Late onset	43.8 (7)	30.4 (7)	50.0 (6)	
History of smoking, % (n)	Nonsmoker	62.5 (10)	73.9 (17)	66.7 (8)	.614
	Smoker	0.0 (0)	4.3 (1)	0.0 (0)	
	Ex-smoker	37.5 (6)	21.7 (5)	33.3 (4)	
Asthma control	Controlled	52.5 (10)	73.9 (17)	66.7 (8)	.385
	Uncontrolled	37.5 (6)	26.1 (6)	33.3 (4)	
ACT score, mean ± SD		21.94 ± 3.90	20.00 ± 5.83	22.33 ± 3.39	.298
No. of asthma exacerbations, median (min-max)		1 (0-2)	1 (0-6)	1 (0-2)	.041∗
Pulmonary function tests, mean ± SD	FEV_1_min %	57.56 ± 19.72	59.56 ± 17.42	47.16 ± 23.97	.210
	FEV_1_min mL	1.32 ± 0.50	1.58 ± 0.59	1.04 ± 0.34	.018†
	FEV_1_/FVC min	63.81 ± 12.35	66.43 ± 11.96	60.00 ± 11.80	.331
	FEV_1_max %	70.50 ± 20.18	76.71 ± 25.47	69.00 ± 33.59	.643
	FEV_1_max mL	1.64 ± 0.53	2.14 ± 0.69	1.56 ± 0.59	.014†
	FEV_1_/FVC max	67.18 ± 11.13	72.43 ± 11.69	62.91 ± 12.63	.074

Group 1: ICS/LABA/LAMA was prescribed at a high dose with reference to ICS; group 2: ICS/LABA/LAMA ± low-dose OCS and added-on biologic therapy during follow-up; group 3: ICS/LABA/LAMA was prescribed at a high dose with reference to ICS ± low-dose OCS/biologic.

A *post hoc* test was performed to identify exactly which groups differed from each other.

*AE,* Allergic eosinophilic; *ANE*, allergic no-eosinophilic; *FEV*_*1*_*min*, minimum FEV_1_; *FEV*_*1*_*max*, maximum FEV_1_; *FVC*, forced vital capacity; *min*, minimum; *max*, maximum; *NAE*, nonallergic eosinophilic; *NANE*, nonallergic no-eosinophilic.

∗ *P* = .028 for the comparison of group 1 and group 2; *P* = .999 for the comparison of group 1 and group 3; *P* = .032 for the comparison of group 2 and group 3.

† *P* = .375 for the comparison of group 1 and group 2; *P* = .508 for the comparison of group 1 and group 3; *P* = .016 for the comparison of group 2 and group 3.

Conclusively, biologic treatment was initiated before ICS/LABA/LAMA in 12 (23.4%) patients in step 5 treatment (group 3). In addition, 117 (76.0%) patients were receiving step 5 treatment without LAMA (*P* < .001). As stated before, patients in group 2 (n = 23) at step 5 were those who initiated biologic treatment after ICS/LABA/LAMA due to asthma exacerbations.

To explore the independent associations between LAMA use and asthma control, we performed an ordinal logistic regression analysis adjusting for GINA treatment step, FEV_1_, eosinophilic phenotype, age, sex, and BMI (Table V). In this model, neither FEV_1_ nor GINA step 5 was significantly associated with poor asthma control, and LAMA use itself was not independently associated with better control after adjustment for these covariates.

**Table V:** Ordinal logistic regression for asthma control (3-level ACT classification), categorized as well controlled, partially controlled, and uncontrolled

Variable	aOR (95% CI)	*P* value
LAMA	1.10 (0.67-1.80)	.710
GINA step	1.31 (0.81-2.12)	.277
FEV_1_ (% predicted)	0.99 (0.98-1.00)	.091
BMI (per unit)	0.99 (0.95-1.03)	.602
Eosinophilic phenotype	0.82 (0.50-1.33)	.418
Age (per year)	1.01 (0.98-1.03)	.532
Sex (female vs male)	0.93 (0.54-1.59)	.779

The model was adjusted for LAMA use, GINA treatment step, lung function (FEV_1_ % predicted), eosinophilic phenotype, age, sex, and BMI.

*aOR*, Adjusted odds ratio.

## Discussion

The role of LAMAs has been studied extensively; however, their effectiveness, particularly in step 4 and step 5 treatment, remains a research area. In this study, we evaluated the use of ICS/LABA/LAMA regardless of demographic characteristics and asthma phenotype in our specialized centers. However, we tend to initiate ICS/LABA/LAMA treatment in patients with lower lung function. In step 4, patients receiving triple therapy showed better asthma control in univariate comparisons. However, this association did not persist after adjusting for confounding variables, suggesting that the observed benefit may reflect baseline differences rather than a causal effect. However, in step 5, regardless of when LAMA was initiated, these patients had more frequent exacerbations and poorer asthma control compared with their counterparts without LAMA, possibly due to differences in asthma severity or treatment selection. Similarly, our study demonstrated a preference for the use of biologics over LAMA in step 5, particularly in dominant type 2 patients, which is consistent with previous studies that indicate that although LAMA offers benefits in moderate asthma, its role in more severe cases requiring biologics is less clear.^12^^,^^13^

Because there were no differences in clinical and demographic characteristics between patients with and without LAMA, this suggests that when initiating ICS/LABA/LAMA it is not crucial to consider age, sex, asthma phenotype, onset of asthma, or smoking. This finding is consistent with the recommendations of existing guidelines.^6^ In addition, we observed that ICS/LABA/LAMA was prescribed in different asthma phenotypes and endotypes, with no significant differences in response, which aligns with previous studies that support this recommendation.^11^^,^^13^, ^14^, ^15^ Unlike biologic therapy, which is selected on the basis of specific inflammatory markers, ICS/LABA/LAMA may be a more versatile option for asthma management.

In our study, the use of ICS/LABA/LAMA in step 4 was associated with better asthma control in univariate comparisons, although this association was no longer statistically significant after adjustment with the ordinal logistic regression model. Previous studies evaluating the efficacy of adding LAMA to a medium dose of ICS/LABA in step 4 have demonstrated reductions in exacerbations and improvements in lung function.^13^^,^^16^ Although our results are directionally consistent with this evidence, they should be interpreted with caution, because the observed benefit may reflect baseline differences rather than an independent effect of LAMA.

In contrast to the GINA recommendations to add LAMA before initiation of biologic therapy in step 5, our study population was already receiving biologic therapy before LAMA was introduced. This is because our centers are allergy and immunology clinics with a high number of patients who are referred for biologic therapy. However, our results may give the perception that ICS/LABA/LAMA can provide sufficient control in moderate asthma while biological therapy is prioritized for more severe cases. Further studies are needed to determine whether this is consistent with different clinical scenarios. Similarly, in a large-scale study with patients with moderate to severe asthma, the use of LAMA or biologic as add-on treatment was evaluated, and it was observed that pulmonologists are more likely to initiate LAMA, in contrast to allergists who tend to initiate biological treatment.^17^ Another explanation for the discrepancy in treatment initiation rates could be that our patients were being followed for an extended period, and the guideline recommendation to initiate LAMA before biologic phenotyping is relatively recent.

Unlike step 4, the use of ICS/LABA/LAMA in step 5 was associated with poor asthma control and more exacerbations. This may be because they were already with a more severe phenotype, which made them more prone to exacerbations, regardless of treatment. In addition, the poor control in these patients can be explained by the lack of biological treatment rather than the ineffectiveness of LAMA. These findings are consistent with other studies in that the initiation of LAMA may be beneficial in severe asthma but less effective than biologics in reducing exacerbations.^18^^,^^19^

The lack of significant association between LAMA use and improved control after adjustment suggests that the apparent benefits observed in step 4 may reflect confounding by disease severity. These results emphasize the importance of phenotyping and adjusting for comorbidities when interpreting the effects of treatment escalation in asthma.

We believe that the present study will make a valuable contribution to the data gap in the use of ICS/LABA/LAMA in patients with asthma, because it is the first study from our country and one of the few real-world studies available in the literature. However, the study also has some limitations. First, because this was a cross-sectional study, no direct results could be obtained to demonstrate the effect of added LAMAs on asthma outcomes. In addition, because of the study design, no follow-up data could be provided. Another limitation is that in our study only data from patients receiving glycopyrronium are presented, because in our country only fixed-dose therapeutic options are available in a single device. In addition, we sought to explore whether the prescribed dose of ICS influences the clinical response to adding LAMA in patients with severe asthma.

Finally, adherence to treatment was not systematically assessed. Forgetfulness or inconsistent use of medications may have significantly affected asthma control outcomes, particularly in patients receiving complex regimens such as triple therapy. This limitation should be addressed in future prospective research.

### Conclusion

In the present study, individuals with asthma receiving step 4 medication with ICS/LABA/LAMA showed better asthma control compared with those without add-on LAMA in univariate analyses; however, this association was no longer statistically significant after adjustment in the ordinal logistic regression model. Our results indicated a preference for biologic treatment over add-on LAMA, especially in type-2–dominant individuals in step 5. Taken together, these findings suggest that ICS/LABA/LAMA may be considered in step 4 patients, whereas in step 5 its role remains less clear given the greater disease severity and frequent use of biologics

**Key messages**

• **Triple therapy showed an apparent benefit in step 4, but this was not statistically significant after adjustment.**
• **No major differences in demographic or phenotypic characteristics were observed between patients who received LAMA and those who did not.**
• **In step 5, patients receiving LAMA add-on therapy had more frequent exacerbations, likely reflecting greater disease severity rather than lack of efficacy.**
• **Biologic therapy appears to be preferred over LAMA in patients with type 2–high asthma in step 5, especially among patients treated in specialized allergy centers.**
• **The findings highlight the need for individualized treatment decisions and underscore the importance of further prospective studies in Latin America.**

## Disclosure statement

This study received partial financial support from AstraZeneca for the publication fee. The sponsors had no role in the design, data collection, analysis, interpretation, or writing of the manuscript.

Statement of AI: During the preparation of this work, the author(s) used ChatGPT (OpenAI) to improve the clarity, grammar, and overall readability of the manuscript. After using this tool, the author(s) reviewed and edited the content as needed and takes full responsibility for the content of the publication.

Disclosure of potential conflict of interest: All the authors declare that they have no relevant conflicts of interest.
